# Impacts of Antibiotic and Bacteriophage Treatments on the Gut-Symbiont-Associated *Blissus insularis* (Hemiptera: Blissidae)

**DOI:** 10.3390/insects7040061

**Published:** 2016-11-03

**Authors:** Yao Xu, Eileen A. Buss, Drion G. Boucias

**Affiliations:** Department of Entomology and Nematology, University of Florida, Gainesville, FL 32611, USA; eabuss@ufl.edu (E.A.B.); pathos@ufl.edu (D.G.B.)

**Keywords:** *Burkholderia*, *Blissus insularis*, midgut crypts, antibiotics, fitness, podovirus

## Abstract

The Southern chinch bug, *Blissus insularis*, possesses specialized midgut crypts that harbor dense populations of the exocellular symbiont *Burkholderia*. Oral administration of antibiotics suppressed the gut symbionts in *B. insularis* and negatively impacted insect host fitness, as reflected by retarded development, smaller body size, and higher susceptibility to an insecticide, bifenthrin. Considering that the antibiotics probably had non-lethal but toxic effects on host fitness, attempts were conducted to reduce gut symbionts using bacteriophage treatment. Soil-lytic phages active against the cultures of specific *Burkholderia* ribotypes were successfully isolated using a soil enrichment protocol. Characterization of the BiBurk16MC_R phage determined its specificity to the Bi16MC_R_vitro ribotype and placed it within the family Podoviridae. Oral administration of phages to fifth-instar *B. insularis*, inoculated with Bi16MC_R_vitro as neonates had no deleterious effects on host fitness. However, the ingested phages failed to impact the crypt-associated *Burkholderia*. The observed inactivity of the phage was likely due to the blockage of the connection between the anterior and posterior midgut regions. These findings suggest that the initial colonization by *Burkholderia* programs the ontogeny of the midgut, providing a sheltered residence protected from microbial antagonists.

## 1. Introduction

Many insect species in the order Hemiptera live on nutritionally deficient food sources (i.e., plant phloem, xylem, seed, or vertebrate blood) that lack essential amino acids, water soluble vitamins (B-vitamins), and/or lipids [1,2]. In many cases, bacterial symbionts such as the *Buchnera* in aphids [1,3], the *Baumannia* in sharpshooters [4], the *Coriobacterium* in pyrrhocorid bugs [5,6], and the *Wigglesworthia* in tsetse flies [7,8] compensate for deficiencies in host’s food sources and play a critical role in host development and survival. However, the nutritional contributions of many gut-symbiotic bacteria in their hemipteran hosts remain unknown. Studies that used antibiotic treatment (ingestion or injection) and/or egg surface sterilization suppressed or eliminated gut symbionts, resulting in increased mortality rates, slower growth, reduced body size, and/or abnormal pale body coloration of their respective host insects [9,10,11,12]. Hence, these bacteria are believed to provide a nutritional role, promote overall fitness (i.e., growth, body size, longevity, fecundity), and/or affect other phenotypic characters (i.e., body coloration) of their hosts [10,13,14]. In addition, gut-symbiotic bacteria have been reported to protect hosts against pathogens [15], parasites [16], and/or xenobiotics [17].

The Southern chinch bug, *Blissus insularis* Barber (Hemiptera: Lygaeoidea: Blissidae), is a plant-phloem-feeding pest of St. Augustinegrass, *Stenotaphrum secundatum* (Walter) Kuntze [18,19]. This insect harbors a dense population of exocellular *Burkholderia* within its tubular midgut crypts [11,20]. Unlike many *Burkholderia*, the *Blissus*-associated *Burkholderia* cannot be directly cultured on bacteriological media but require an initial organ culture to transition these bacteria to an in vitro environment [20]. Genomic analysis has revealed that most *B. insularis* individuals harbor a single *Burkholderia* ribotype while a chinch bug population harbors a diverse *Burkholderia* community [20,21]. A recent study reveals that the oral acquisition of *Burkholderia* from the environment plays a key role in maintaining ribotype diversity of gut symbiont in *B. insularis* populations [21]. In addition to *B. insularis*, exocellular *Burkholderia* symbionts are also found in the midgut lumen of heteropterans in other genera of Lygaeoidea and Coreoidea superfamilies [22,23,24,25]. These crypt-associated *Burkholderia* may be orally acquired by each generation from the environment as demonstrated in *Riptortus pedestris* (F.) [10], or be acquired both from symbiont-contaminating eggs and from the environment as shown in *Cavelerius saccharivorus* Okajima [23]. *Burkholderia* spp. also are detected in the bacteriomes of a leafhopper, *Macrosteles striifrons* Anufriev [26], and in the fat body cells and ovaries of two scale insect species, *Gossyparia spuria* (Modeer) and *Acanthococcus aceris* Signoret [27]. Recently, a transovarial transmission mechanism of *Burkholderia* symbiont is revealed in these scales insects, indicating a migration of *Burkholderia* cells from the body cavity to the developing oocyte for ensuring the infection of obligatory symbionts in host offspring [27].

In a previous study, first-instar *B. insularis* that were reared on antibiotic-coated St. Augustinegrass had fewer *Burkholderia* 16S rRNA gene copies in the midgut crypts, exhibited lower survivorship and slower development than insects fed antibiotic-free grass. These findings suggested that gut-symbiotic *Burkholderia* assisted in the fitness of *B. insularis* [11]. However, the information related to the biological function of *Burkholderia* in *B. insularis* is limited. In addition to being the primary pest of St. Augustinegrass lawns, *B. insularis* is notorious for its rapid development of resistance against insecticides, including bifenthrin, the predominant one used currently against *B. insularis* in Florida [28,29,30]. The mechanism(s) underlying the insecticide resistance in *B. insularis* remains unknown. The reported gut-symbiont-mediated insecticide resistance in the bean bug *R. pedestris* [17] stimulated a closer examination of the potential role of *Burkholderia* in the fitness of *B. insularis*.

According to the ribotyping, many gut-symbiotic *Burkholderia* in *B. insularis* are closely related to the free-living *Burkholderia* species that inhabit soils [11,20]. The soil bacterial community contains an estimated 1 × 10^7^ bacteriophages per gram of soil [31]. From soils, bacteriophages that lyse soil-borne *Burkholderia pseudomallei* and other virulent *B. cepacia* complex (Bcc) species have been isolated and tested as biological controls targeting infectious disease agents [32,33]. Such lytic bacteriophages are highly specific to target bacterial species, are capable of rapid replication, and are innocuous to eukaryotes [34]. For example, the soil-derived *Burkholderia* phage, applied as an antibacterial therapy for the Bcc lung infections, has shown in vivo efficacy in reducing *Burkholderia* density and the lung inflammation [35]. Using soil-derived bacteriophage that targeted the gut-symbiotic *Burkholderia*, oral delivery of bacteriophage to *B. insularis* was tested as an alternative approach to antibiotic treatment. Overall, the impacts of antibiotic and bacteriophage treatments on the gut-symbiotic *Burkholderia* and on the fitness of host *B. insularis* were examined in the current study.

## 2. Materials and Methods

### 2.1. Insect Rearing and Administration of Antibiotics

**Figure 1:**
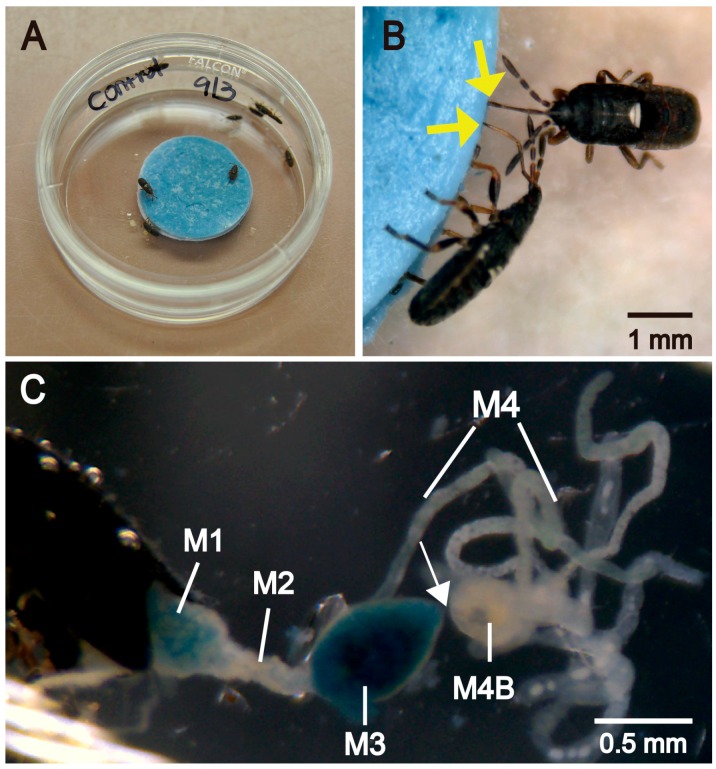
Oral delivery of antibiotics to *Blissus insularis*. (**A**) Fifth instars were placed into a feeding arena with liquid food supplemented with Evans Blue dye and antibiotics; (**B**) Fifth instars were feeding. Yellow arrows indicate the mouthparts of feeding *B. insularis*; (**C**) Dissected digestive tracts from fifth instar *B. insularis* after 10-day exposure to the antibiotic treatments. Abbreviations: M1, midgut first section; M2, midgut second section; M3, midgut third section; M4, midgut fourth section with crypts; M4B, M4 bulb. Note the presence of dye in the M3, but not M4B or M4 section. Arrow indicates the thin, thread-like constriction connecting the M3 and M4B sections.

Fourth-instar *B. insularis*, collected from St. Augustinegrass, were pooled, placed in a plastic container, and provisioned with St. Augustinegrass every two days until they molted into fifth instars. Preliminary attempts failed to keep delicate neonates and young instars alive in the oral administration of antibiotics experiment [36]. Therefore, the more robust fifth instars were used to ensure the feasibility of the experiment. Fresh corn kernels, which were used to rear *B. insularis* [37], were ground manually with a mortar and pestle and centrifuged at 12,000× *g* for 10 min. Supernatants were supplemented with 0.02% of the lipophilic Evans Blue dye (MW: 961, Sigma-Aldrich, St. Louis, MO, USA) to visualize food passage into the digestive tract of *B. insularis*. Two water-soluble antibiotics (oxytetracycline and kanamycin) that effectively inhibited various gut-symbiotic *Burkholderia* isolates were selected [20], made separately with two concentrations (1.4 and 4 mM) and two exposure times (five and 10 days) prior to the feeding assays to ensure that the optimized antibiotic treatment (1.4 mM, 10 days) reduced crypt-inhabiting *Burkholderia* with minimal lethality on the host *B. insularis* [36]. The corn supernatants with dye were amended with 1.4 mM of antibiotics and applied to sterile, three-layered, circular glass microfiber filter paper discs (1.6 cm diameter, Whatman Inc., Clifton, NJ, USA). Saturated discs were placed in a sterile petri dish (35 × 10 mm, Falcon^®^, Corning Inc., Corning, NY, USA) containing five to ten newly molted fifth instars (Figure 1A). Test insects were exposed for 10 days to liquid food supplemented with a daily rotation of oxytetracycline or kanamycin. As controls, fifth instars were provisioned with antibiotic-free liquid food. Each treatment was replicated seven times. A total of 63 and 66 fifth-instar *B. insularis* were used in the antibiotic-treated and control groups, respectively.

### 2.2. Impacts of Antibiotic Treatment on Host B. insularis

During the 10-day exposure to either the antibiotic-treated or control food, *B. insularis* survival and adult eclosion rates were recorded. The Kolmogorov-Smirnov test (PROC UNIVARIATE, SAS 9.3; SAS Institute Inc., Cary, NC, USA) indicated that neither the survivorship data (N = 14, D = 0.2563, *p* = 0.014) nor the adult eclosion rate data (N = 14, D = 0.3347, *p* < 0.010) were normally distributed. Therefore, survivorship and the adult eclosion rate were subjected separately to the Wilcoxon Two-sample Tests (PROC NPAR1WAY, SAS 9.3) for comparing the difference between the antibiotic-treated and control groups. The body length of fifty antibiotic-treated and 36 control unsexed fifth instars were compared using the two-sample *t*-test (PROC TTEST, SAS 9.3). Consequently, a total of 33 antibiotic-treated and 20 control fifth instars were subjected to the quantitative PCR (qPCR) analysis to estimate the copy numbers of *Burkholderia* 16S rRNA gene in their respective midgut crypts. Dissected crypts were surface-sterilized individually by immersion for three min each in 70% ethanol (EtOH), 5% bleach, and 70% EtOH. The presence of Evans Blue dye in the dissected digestive tracts served as a relative indicator of food ingestion. At dissection, the presence of testes and ovaries indicated the sex of the assayed fifth instars. Dissected intact midgut crypts were rinsed at least three times in sterile H_2_O and subjected to DNA extraction using the MasterPure™ Yeast DNA Purification Kit (Epicentre, Madison, WI, USA) [20].

### 2.3. Susceptibility of Antibiotic-Treated B. insularis to Bifenthrin

A contact bioassay was used to assess the relative susceptibility of antibiotic-treated and control *B. insularis* fifth instars to bifenthrin (Talstar P^®^, FMC Corp., Philadelphia, PA, USA) [38]. Dilutions of bifenthrin (0.1 μg·mL^−1^) or acetone (solvent control) were added into clean 16-mL glass vials (0.5 mL per vial), and then rotated on a roller for one hour to evaporate the solvent carrier. Coated vials were plugged with moistened cotton balls, inoculated with five to ten unsexed fifth instars, held at room temperature (RT) in darkness, and examined after 24 h to determine the percentage of paralyzed insects. The paralysis rates of the antibiotic-treated and control insects were normally distributed (N = 14, D = 0.1537, *p* > 0.1500) according to the Kolmogorov-Smirnov test and therefore were compared using the two-sample *t*-test (PROC TTEST, SAS 9.3). A total of 17 paralyzed and 45 non-paralyzed *B. insularis* fifth instars that were exposed to bifenthrin in the contact bioassays were subjected to the qPCR analysis.

### 2.4. Quantitative PCR on DNA Preparations

The copy numbers of the *Burkholderia* 16S rRNA gene in genomic DNA samples (see Section 2.2 and Section 2.3) were estimated by qPCR. The in vitro *Burkholderia* isolate (Bi16MC_R_vitro, ATCC deposit number: TSD-41) served as the external standard in the qPCR analysis. Approximately 6 × 10^8^ colony-forming units (CFU) of the external standard, estimated by plating the culture on a nutrient agar (0.3% beef extract, 0.5% peptone, 1.5% agar; Becton, Dickinson and Company, NJ, USA) plate, were subjected to DNA extraction using the MasterPure™ Yeast DNA Purification Kit. Dilutions of the extracted DNA (10–10^7^ copy number equivalents) were subjected to qPCR reactions [11] to generate a strand curve. The Ct values of genomic DNA samples extracted from assayed insects were estimated from the standard curves and converted into the copy numbers of *Burkholderia* 16S rRNA gene per insect. Copy numbers were log_10_-transformed before statistical analysis, and the normal distribution was confirmed with the Kolmogorov-Smirnov test (N = 62, D = 0.0896, *p* > 0.1500). The log_10_-transformed copy numbers were compared separately between sexes, antibiotic treatments, as well as responses to bifenthrin exposure (paralyzed and non-paralyzed), using the two-sample *t*-test (PROC TTEST, SAS 9.3).

### 2.5. Histology

After 10 days of exposure to the antibiotic-treated or control food, dissected midgut crypts from fifth instar *B. insularis* were placed on a pre-cleaned Gold Seal^®^ (Gold Seal Products, Portsmouth, NH, USA) Fluorescent Antibody RITE-ON microslide, and stained directly with SYTO 9 dye and propidium iodide (LIVE/DEAD^®^ BacLight™ Bacterial Viability Kit, Molecular Probes, Eugene, OR, USA) at RT in darkness for 15 min. Stained crypts were washed three times in HEPES (10 mM, pH 7.4), mounted in 1,4-diazabicyclo[2.2.2]octane in glycerol (DABCO), and examined using epifluorescence microscopy. Second, dissected crypts were fixed in 4% paraformaldehyde at 4 °C overnight, washed three times in HEPES buffer, incubated with 1 µg mL^−1^ of DAPI at RT in darkness for 10 min, washed, mounted in DABCO, and examined using the epifluorescent optics. Third, dissected crypts were fixed in 2.5% glutaraldehyde at 4 °C overnight, post-fixed in 1% aqueous osmium tetroxide, dehydrated in a graded ethanol series, and infiltrated in LR White medium epoxy resin and Z6040 embedding primer (Electron Microscopy Sciences, Hatfield, PA, USA). Sections collected on carbon-coated copper grids were stained with 2% uranyl acetate and Reynold’s lead citrate [39]. Sections were examined with a FEI Tecnai G2 Sprit Twin transmission electron microscope (FEI Corp., Hillsboro, OR, USA). Digital images were acquired with an AMT-ER41 1 k × 1 k camera with TIA software (FEI Corp.) and a Gatan UltraScan 2 k × 2 k camera with the Digital Micrograph software (Gatan Inc., Pleasanton, CA, USA).

### 2.6. Isolation and Purification of Gut-Symbiotic Burkholderia Phages

An enrichment method for phage isolation [40] was conducted using soils collected from ten St. Augustinegrass lawns. Eleven *Burkholderia* cultures isolated from the crypts of *B. insularis* females were used as substrates to amplify soil bacteriophage populations. Approximately 5 g of pooled soil samples from each lawn were inoculated into 15 mL of nutrient broth medium (0.3% beef extract, 0.5% peptone), mixed with 0.5 mL of mid-log phase *Burkholderia* cultures (5 × 10^8^ cells mL^−1^), and incubated at 200 rpm, 28 °C for 14–16 h. Subsequently, 100 µL of the enriched culture was centrifuged at 10,000× *g*, 4 °C for 15 min, and supernatants were filtered through a 0.45-µm filter. Filtrates were screened for lytic phage activity using a modified spot-on-the-lawn technique [41]. Nutrient agar plates were flooded with mid-log phase bacterial cultures and incubated at RT for 10 min. Excess culture fluid was removed and dried for 25 min in a sterile flow hood. Five microliters of soil filtrate from each enriched sample were spotted onto the lawn and incubated at 28 °C overnight. In order to examine the specificity of detected lytic bacteriophages, soil filtrates were screened against a series of *Burkholderia* isolates. A total of 11 *Burkholderia* cultures were selected as bacterial lawns. Lytic phage activity was indicated by a clear zone or plaque formation. Lytic bacteriophages were picked from the clear zones of spotted nutrient agar plates for all tested soil filtrates, and subsequently they were cloned and amplified using the top-agar overlay method [42]. For each phage, cloning of plaques was repeated at least twice to ensure the homogeneity of the phage stock, which was prepared by plate lysis and elution method [42]. Bacteriophage preparations were tittered using the top-agar overlay method.

### 2.7. Characterization of a Selected Burkholderia-Lytic Phage

A soil-derived, purified bacteriophage termed BiBurk16MC_R phage that lysed the gut-symbiotic *Burkholderia* isolate (Bi16MC_R_vitro) was subjected to TEM. The phage suspension (2 × 10^10^ PFU∙mL^−1^) was floated onto the carbon-coated Formvar on 400 mesh copper grid (Electron Microscopy Sciences) for 5 min. Grids were stained with 1% aqueous uranyl acetate and examined with a Hitachi H-7000 TEM (Hitachi High Technologies America, Inc. Schaumburg, IL, USA). Digital images were acquired with a Veleta 2 k × 2 k camera with iTEM software (Olympus Soft-Imaging Solutions Corp., Lakewood, CO, USA). Bacteriophage size was determined from the average of four independent measurements.

Purified BiBurk16MC_R phage suspension (2 × 10^10^ PFU∙mL^−1^) was treated with 500 units of Benzonase^®^ nuclease (Sigma-Aldrich) for one or two hours to remove potential host DNA contaminants. The nuclease-treated phage suspension was subjected to nuclei acid extraction using the MasterPure™ Yeast DNA Purification Kit. Approximately 300 ng of the extracted phage nucleic acid were digested with *BamHI*, *EcoRV*, *HindIII*, *PstI*, *PvuII*, or *XbaI* restriction endonucleases (REN, Promega, Madison, WI, USA). The genome size of double-stranded DNA was estimated by Pulsed Field Gel Electrophoresis (PFGE). Twenty microliters of the REN digests were mixed with 5× loading dye and loaded into a 1% Pulsed Field Certified Agarose gel in 0.5× TBE. Electrophoresis, conducted at 14 °C, was run for four hours at 6 V∙cm^−1^ with 0.1 s switch time. Standard markers were Lambda DNA/*EcoRI* plus *HindIII* and 100-bp molecular ruler (Bio-Rad Laboratories, Hercules, CA, USA). The PFGE patterns were visualized after staining with 1× SYBR Gold Nucleic Acid Gel Stain (Molecular Probes, Eugene, OR, USA). Fragment sizes were estimated based on the standard molecular weight using the Quantity One software (Bio-Rad).

### 2.8. Oral Delivery of Bacteriophage to B. insularis

Due to the high specificity of bacteriophage to the target bacteria, *B. insularis* infected with target gut-symbiotic *Burkholderia* were generated by rearing neonates on live plants inoculated with cultured symbionts (Bi16MC_R_vitro isolate), as described previously [21]. After three weeks of rearing on *Burkholderia*-inoculated plants, fourth instars were collected, provided with surface-sterilized corn kernels in a plastic container, and held at 27 °C with a 14:10 (L:D) h photoperiod. Newly molted fifth instars (<24-h old), presumed to harbor the inoculated Bi16MC_R_vitro, were exposed to the BiBurk16MC_R phage. The four treatments included food supplemented with phage particles, Bi16MC_R_vitro culture, phage-infected Bi16MC_R_vitro culture, or nutrient broth (blank control). The food substrate consisted of corn juice, 0.02% Evans Blue dye, and 2 mM CaCl_2_. For treatment with phage particles, 5 μL of purified phage stock suspension (2 × 10^9^ PFU∙mL^−1^) in TBS were added to 245-μL food for loading onto the glass microfiber filter paper. For the treatment with Bi16MC_R_vitro culture, 5 μL of overnight-cultured Bi16MC_R_vitro in nutrient broth medium (~5 × 10^8^ cells mL^−1^) was added. For treatment with phage-infected Bi16MC_R_vitro culture, both purified phage suspension (5 μL) and overnight-cultured Bi16MC_R_vitro (5 μL) were added. Fifth instars were exposed to each treatment for 10 days. Diet-treated discs were prepared and replaced daily. Treatments were replicated five times. At the end of 10 days of exposure, survival and adult eclosion rates were recorded. The Kolmogorov-Smirnov test indicated that neither survival (N = 20, D = 0.5065, *p* < 0.0100) nor adult eclosion rate (N = 20, D = 0.1700, *p* = 0.1320) data was distributed normally. Therefore, the Kruskai-Wallis test (PROC NPAR1WAY) was used for comparing the difference in mean values between four diet treatments.

To determine whether or not the BiBurk16MC_R phage was ingested by *B. insularis*, three to five survivors from each diet treatment were surface-sterilized and their midguts were dissected. The posterior (M4B and M4) and anterior (M1-M3) midgut sections were dissected and homogenized in 150 μL of TBS using a sonic dismembrator (model 300; Fisher Scientific). Aliquots (100 μL) of the posterior midgut homogenates were extracted with the MasterPure™ Yeast DNA Purification kit. The remaining homogenates amended with 2 mM CaCl_2_ were subjected to the spot-on-the-lawn technique to estimate PFUs. Purified BiBurk16MC_R phage (~2 × 10^7^ PFU∙mL^−1^) was used as a positive control. The Kruskai-Wallis test (PROC NPAR1WAY, SAS 9.4) compared the difference in mean values between four diet treatments, due to the non-normally distributed percentage of positive plaque formation data (N = 20, D = 0.3250, *p* < 0.0100). A post-hoc test of DSCF was applied for pairwise two-sided multiple comparison analysis.

To confirm the rate of *B. insularis* infected with the inoculated Bi16MC_R_vitro, genomic DNA from posterior midgut homogenates were subjected to BOX-PCR fingerprinting using a BOX-A1R primer [43], as modified previously [20]. Samples producing ≥75% homology in the BOX pattern of that produced by the Bi16MC_R_vitro DNA were considered as positive infections [21].

## 3. Results

### 3.1. Impacts of Antibiotic Treatment on Host B. insularis

During the 10-day exposure to food supplemented with a rotation of antibiotics, fifth instars of *B. insularis* probed their stylets into the food-containing filter paper discs (Figure 1B) and remained immobile for up to 10 min during individual feeding events. Dissection after 10 d revealed the presence of Evans Blue dye in the anterior midgut regions (M1-M3), but dye was not detected in the posterior regions (M4B-M4) where bacterial symbionts are localized (Figure 1C). This observation was found in all examined *B. insularis*, regardless of sex, indicating that the rotating antibiotics were ingested and entered the anterior midgut regions.

**Figure 2:**
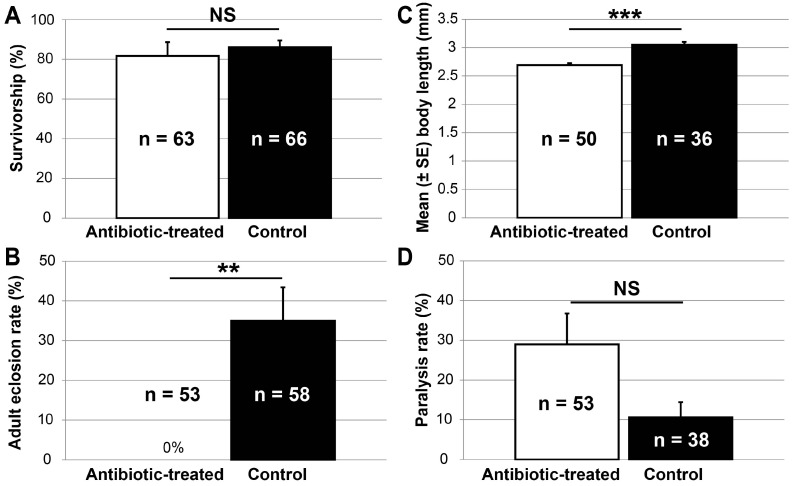
Impacts of 10-day exposure to the antibiotic-treated and the control diet (corn juice) on the mean (±SE) *Blissus insularis* survivorship (**A**); adult eclosion rate (**B**); body length (**C**); and paralysis rate at 24-h exposure to bifenthrin (**D**). The number in each bar indicates the total number of *B. insularis* fifth instars that were subjected to each treatment. NS, no significant difference (*α* = 0.05); ** *p* < 0.01; *** *p* < 0.001.

At the end of 10-day antibiotic treatment, 82% ± 7% and 86% ± 3% (±SE) of the examined antibiotic-treated and control *B. insularis*, respectively, survived. No difference in the survivorship was found between these two groups (normal approximation; z = 0.2570, *p* = 0.7972) (Figure 2A). However, none of the 53 antibiotic-exposed *B. insularis* eclosed to the adult stage, in contrast to the 31% (18/58) of the control insects (Figure 2B). The male-to-female sex ratio among these adults was 2:1. In addition to impacting adult eclosion, the mean (±SE) body length of antibiotic-treated fifth instars (*n* = 50, 2.7 ± 0.03 mm) was significantly smaller (*t* = 6.18, df = 57.61, *p* < 0.001) than that of control fifth instars (*n* = 36, 3.1 ± 0.05 mm) (Figure 2C). The delayed adult eclosion and reduced body length suggested that antibiotic treatment retarded *B. insularis* growth.

### 3.2. Impacts of Antibiotic Treatment on Burkholderia 16S rRNA Gene Copy Number

**Figure 3:**
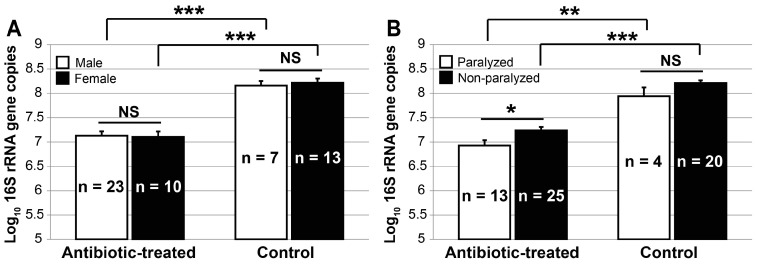
The estimated mean (±SE) log_10_ *Burkholderia* 16S rRNA gene copies per insect in the midgut crypts of *Blissus insularis* fifth instars, from the antibiotic-treated and control groups. (**A**) Mean (±SE) log_10_ *Burkholderia* 16S rRNA gene copies in the male and the female fifth instars; (**B**) Mean (±SE) log_10_ *Burkholderia* 16S rRNA gene copies in the paralyzed and the non-paralyzed fifth instars that were exposed to 0.1 μg·mL^−1^ of bifenthrin. The number in each bar indicates the total number of *B. insularis* fifth instars within each treatment. NS, no significant difference (*α* = 0.05); * *p* < 0.05; ** *p* < 0.01; *** *p* < 0.001. See tabulated data in Table S1.

The DNA preparations extracted from the antibiotic-treated fifth instars contained an estimated 1.9 ± 0.2 × 10^7^ (±SE) *Burkholderia* 16S rRNA gene copies per insect. This number was significantly less (*t* = 11.34, df = 60, *p* < 0.0001) than the 1.8 ± 0.2 × 10^8^ *Burkholderia* 16S rRNA gene copies in the control preparations. The reduction in copy numbers was not attributed to the difference in sex ratios of examined fifth instars. In the antibiotic-treated group, the estimated *Burkholderia* 16S rRNA gene copies per female (1.6 ± 0.3 × 10^7^) were not significantly different (*t* = 0.16, df = 31, *p* = 0.8732) from those of male fifth instars (1.9 ± 0.3 × 10^7^). Likewise, in the control group, the *Burkholderia* 16S rRNA gene copies per female (2.1 ± 0.4 × 10^8^) were not significantly different (*t* = 0.47, df = 18, *p* = 0.6434) from those of male fifth instars (1.6 ± 0.3 × 10^8^) (Figure 3A). Male and female groups from the antibiotic group harbored 9-fold and 13-fold fewer gene copies, respectively, than did their control counterparts.

### 3.3. Histological Examinations of Midgut Crypts

The dissected digestive tracts of control insects contained milky-white and thick crypts, whereas the crypts of antibiotic-treated insects were semi-transparent and slender. Vital staining with LIVE/DEAD BacLight Kit revealed that many crypt-inhabiting bacteria of the antibiotic-treated insects fluoresced red, indicating that they were dead. Crypts from control insects contained a high number of green signals (live bacteria) and relatively few red signals (Figure S1). Excluding the host cell nuclear regions, the DAPI-stained and fixed crypts of control insects produced stronger fluorescent signals than those of antibiotic-treated insects (Figure S2). The increased number of signals in control group was likely due to the crypt-inhabiting bacteria.

**Figure 4:**
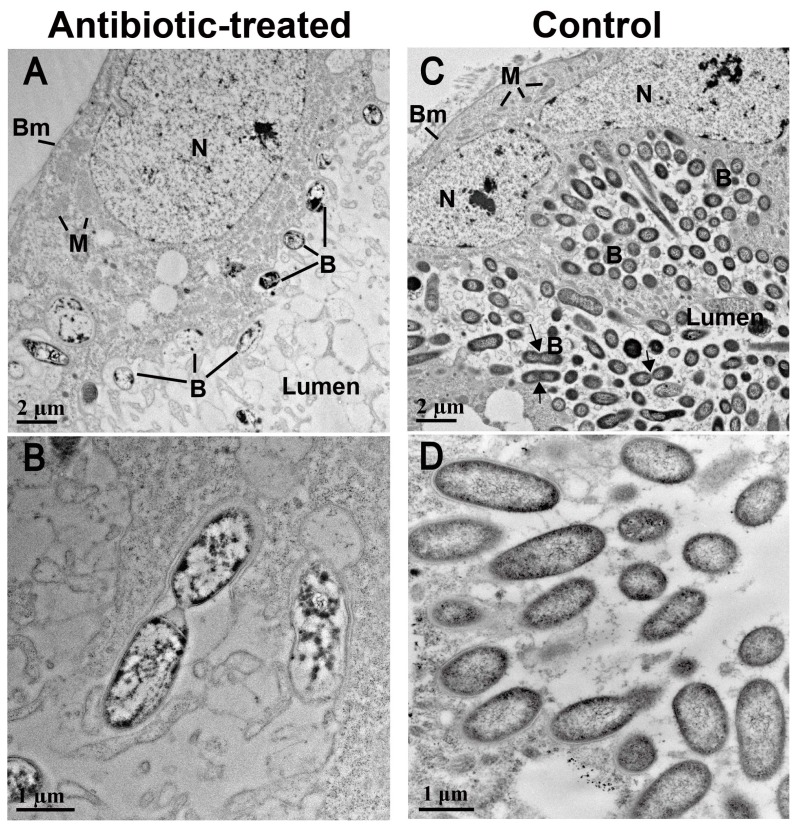
Transmission electron microscopy of midgut crypts dissected from the antibiotic-treated and control *Blissus insularis* fifth instars. (**A**) The crypts of antibiotic-treated *B. insularis*. Only a few rod-shaped bacteria (denoted as B) were present in the crypt lumen; (**B**) The enlarged micrograph of bacteria exhibiting abnormal shape; (**C**) The crypts of control *B. insularis*. Numerous bacteria were present in the crypt lumen and were dividing by binary fission (indicated by arrows); (**D**) The enlarged micrograph of bacteria exhibiting normal shape. Abbreviations: B, symbiotic bacterium; Bm, host crypt basement membrane; N, host crypt nucleus; M, host crypt mitochondrion.

TEM observations showed that crypt lumens of control *B. insularis* were filled with numerous rod-shaped bacteria measuring approximately 2.20 ± 0.12 μm by 0.82 ± 0.04 μm. In several ultra-thin sections, actively dividing bacterial cells were observed within the control crypts (Figure 4C). Conversely, the 10-day exposure to antibiotic-treated food eliminated the majority of bacteria inhabiting the crypt lumen. Only a few rod-shaped, intact bacteria with well-developed cell walls were found in multiple sections of the antibiotic-treated crypts (Figure 4A). Measurement of these bacteria was 2.89 ± 0.21 μm by 1.02 ± 0.04 μm. Moreover, the lumen of antibiotic-treated crypts was typically filled with material resulting from bacterial lysis; no such material was found in the control crypts (Figure 4D). In the antibiotic-treated crypts, distorted bacteria with electron-dense cellular structures were observed, implying that these bacteria were dead (Figure 4B). The antibiotic-induced elimination of microbiota in crypt lumen did not induce detectable cytopathology on the host crypt cells.

### 3.4. Susceptibility of Antibiotic-Treated B. insularis to Bifenthrin

Due to the retarded development of *B. insularis* resulting from the 10-day antibiotic treatment, more antibiotic-treated (*n* = 38) than control (*n* = 24) fifth instars were available for the contact bioassay. After 24 h of exposure to bifenthrin-coated vials, the paralysis rate of antibiotic-treated *B. insularis* fifth instars was 29% ± 8%, a rate approximately three-fold higher, but not significantly different (*t* = 1.96, df = 12, *p* = 0.0737) from the 11% ± 4% control paralysis rate (Figure 2D). As reported in prior assays, within a treatment, the *Burkholderia* 16S rRNA gene copies generated from females were not significantly different from those of males (Figure 3A). Consequently, the copy numbers of both sexes were pooled together within each treatment. In the control group, the mean log_10_ *Burkholderia* 16S rRNA gene copies per insect between four paralyzed and 20 non-paralyzed individuals were not significantly different (*t* = 1.88, df = 22, *p* = 0.0738) (Figure 3B). Specifically, in the control group, paralyzed and non-paralyzed fifth instars harbored 1.2 ± 0.6 × 10^8^ and 1.9 ± 0.3 × 10^8^ *Burkholderia* 16S rRNA gene copies per insect, respectively (Supporting Information, Table S1). After the 10-day exposure to the antibiotic-treated food, the 13 paralyzed fifth instars harbored 1.2 ± 0.3 × 10^7^ *Burkholderia* 16S rRNA gene copies per insect, which were significantly less (*t* = 2.54, df = 36, *p* = 0.0155) than the 25 non-paralyzed insects that contained 2.2 ± 0.3 × 10^7^ copies per insect (Figure 3B). Furthermore, for the paralyzed fifth instars, the mean log_10_ *Burkholderia* 16S rRNA gene copy numbers of the antibiotic-treated *B. insularis* was approximately 10-fold less than those of the control counterparts (*t* = 4.66, df = 15, *p* = 0.003). Similarly, for the non-paralyzed fifth instars, the mean log_10_ *Burkholderia* 16S rRNA gene copy numbers of the antibiotic-treated fifth instars was 9-fold less than those of the control ones (*t* = 10.52, df = 43, *p* < 0.001).

### 3.5. Isolation and Screening of Burkholderia-Lytic Phages

Preliminary studies demonstrated that the enrichment method efficiently amplified and isolated the *Burkholderia*-lytic phages from soils. Specifically, the lytic phage activities of *Burkholderia*-enriched soil filtrates were approximately eight times higher than those of unenriched filtrates (Figure S3). Five of 11 examined *Burkholderia*-enriched soil filtrates exhibited specific phage activities against their respective gut-symbiotic *Burkholderia* in vitro (Table S2). Three enriched soil filtrates had lytic activities against multiple *Burkholderia* isolates, whereas the remaining three enriched filtrates exhibited no activity against *Burkholderia*. 

### 3.6. Characterization of a Selected Burkholderia-Lytic Phage

**Figure 5:**
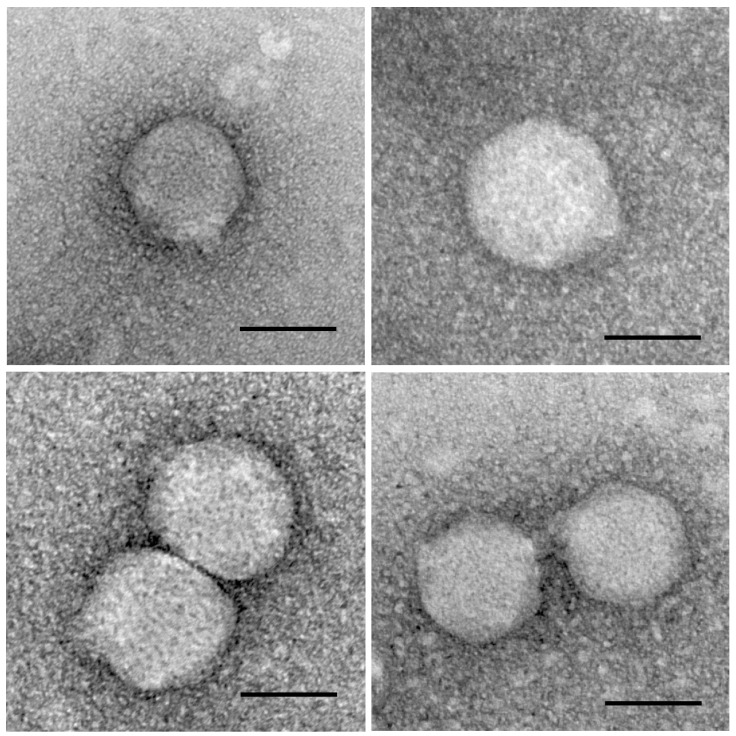
Transmission electron micrographs of the negative-stained gut-symbiotic *Burkholderia* phage BiBurk16MC_R, photographed in four different fields. Scale bars indicate 50 nm.

Transmission electron microscopy revealed that the BiBurk16MC_R phage contained an isometric head measuring 73 ± 0.9 nm in diameter, and a short tail measuring 10 ± 0.4 nm in length and 15 ± 0.3 nm in width (Figure 5). On the top agar plate against the Bi16MC_R_vitro bacterial culture, the phage produced clear plaques (1–2 mm in diameter). The Benzonase^®^ nuclease-treated undigested phage DNA for one and two hours yielded similar bands in intensity on the gel (Figure S4). Therefore, a 1-h nuclease treatment was applied to the phage suspension before digestion using REN. Attempts to digest phage DNA using six different REN only succeeded with the enzyme *EcoRV*, producing 12 separated bands on the gel; whereas other five REN digests resulted in an increased mobility, compared to undigested DNA (Figure S4). These observations indicated that the phage preparation contained open circular double-stranded viral DNA with single restriction sites for *BamHI*, *HindIII*, *PstI*, *PvuII*, and *XbaI* enzymes. In addition, digestion with the enzyme *PvuII* produced a smear on agarose gel (Figure S4). Based on the PFGE analysis of the *EcoRV* digest, the estimated genome size of this phage was 45.8 kb (Figure S4). The morphological characterizations and double-stranded DNA indicated that this phage belongs to family Podoviridae, according to the guidelines of the International Committee on Taxonomy of Viruses (http://www.ictvonline.org/index.asp).

### 3.7. Oral Delivery of Bacteriophage to B. insularis

After 10-day exposure to the food supplemented with BiBurk16MC_R phage, 27 of 28 examined *B. insularis* survived, and eight eclosed to the adult stage. In the treatment with phage-infected *Burkholderia*, all 29 examined *B. insularis* survived with five eclosing to adults. For the treatments containing no phage (*Burkholderia* only and control groups), 96% and 95% survived, and 39% and 34% eclosed to adults, respectively. Within each treatment, the female to male ratio of eclosed adults was approximately 1:1. No difference was found in the survivorship (*χ*^2^ = 1.1385, df = 3, *p* = 0.7678) or in the adult eclosion rate (*χ*^2^ = 2.3887, df = 3, *p* = 0.4957) among the four treatments. Regardless of the treatment, dissection of 97 surviving *B. insularis* adults and fifth instars revealed that all insects had blue dye in the anterior midgut regions (M1-M3). Similar to the findings in the antibiotic assays, no blue dye appeared in the posterior midgut regions (M4B-M4) or in the hindgut. The BOX-PCR results on the DNA preparations of insects sampled from the four treatments revealed that approximately 20%–50% of the preparations harbored the inoculated Bi16MC_R_vitro (Table 1).

In the phage treatment, 20 of 25 anterior homogenate samples exhibited positive phage activities; however, no plaque activity was observed in their respective posterior homogenates. In the phage-infected *Burkholderia* treatment, 23 of 25 examined anterior homogenate preparations had positive phage activities. Again, no plaques were produced in the phage screening of the posterior homogenates. In the treatments containing no phage (*Burkholderia* only and control groups), neither the anterior nor the posterior midgut homogenate produced plaques (Table 1). In all tested plates, clear zones were detected in positive controls, whereas no plaque appeared in negative controls (TBS buffer) (Figure S5). Again, it should be noted that 40% and 50% of the individuals fed the phage-containing diet (phage and phage-infected *Burkholderia* groups, respectively) were confirmed to harbor the inoculated target Bi16MC_R_vitro isolate (Table 1).

## 4. Discussion

In a previous study, the 11-day exposure of *B. insularis* first instars to tetracycline-treated St. Augustinegrass stolons induced a 7-fold reduction of *Burkholderia* 16S rRNA gene copies in the midgut crypts, compared to the insects that were fed on the antibiotic-free stolons [11]. In the current study, the quantitative estimation of *Burkholderia* 16S rRNA gene copies per antibiotic-treated *B. insularis* fifth-instar insect was 10-fold less than the copy numbers detected in control crypts. The qPCR-generated gene copy numbers of gut-symbiotic bacteria likely overestimate bacterial numbers. The TEM examination of crypts that were exposed to antibiotic treatment depicted massive removal/killing of crypt bacteria (Figure S2). This situation is reminiscent of the discrepancy between CFU counts and qPCR results in *R. pedestris* [44], suggesting that most 16S rRNA gene copies in the antibiotic-treated crypts detected by qPCR were amplified from the dead symbiont DNA contents. It should be noted that the *Blissus* crypt-associated *Burkholderia*, unlike the *R. pedestris* crypt-associated *Burkholderia*, cannot be directly plated onto bacteriological media for CFU counts [20]. Typically, bacterial 16S rRNA gene copies estimated by qPCR are good indictors for bacterial mass but not for bacterial viability [45].

Our data did demonstrate that exposure to antibiotics suppressed the growth and development of *B. insularis*. Indeed, it has been reported that such antibiotic induced suppression of bacterial symbionts may negatively impact host fitness. For example, the oral delivery of rifampicin eliminated the transovarially transmitted endocellular *Nardonella*, and this antibiotic treatment retarded the growth and development of the host weevil, *Euscepes postfasciatus* (Fairmaire) [46]. Significantly, the offspring of antibiotic-treated adults maintained on an artificial diet developed more slowly than the offspring of parents fed an antibiotic-free diet. Similar observations were made with the hematophagous bed bug, *C. lectularius*. This species exhibited a prolonged nymphal period with a low adult eclosion rate after it was continuously reared on antibiotic-supplemented meals [47]. The involvement of symbionts in bedbug nutrition was demonstrated by feeding nymphs antibiotic-spiked blood supplemented with vitamins. The addition of vitamins neutralized the negative impacts of the loss of symbionts.

Antibiotic-treated *B. insularis* fifth instars were 3-fold more susceptible to bifenthrin exposure than were controls. Furthermore, within the antibiotic-treated group, paralyzed insects that were susceptible to bifenthrin exposure had fewer *Burkholderia* 16S rRNA gene copies than the non-paralyzed insects. These results suggest that the antibiotic treatment directly or indirectly impacts the susceptibility of *B. insularis* to bifenthrin. Likely, the increased susceptibility is due to antibiotic effect on *B. insularis* fitness; it may be assumed that this is a result of the suppression of crypt-inhabiting *Burkholderia*. In addition to impacting the growth and development of the host insect, perturbations in the insect-associated microflora are known to impact host defense. Many insects rely on their obligate endosymbionts to maintain their innate defense systems to protect them from a range of environmental challenges [17,48,49,50,51].

One cannot rule out the possibility that the antibiotic therapy used to eliminate *Burkholderia* exhibited sub-lethal toxicity to *B. insularis*. Specifically, the tetracycline class of antibiotics, in addition to having potent antibacterial activities, has other secondary toxic effects to eukaryotes. It has been pointed out that eukaryotic mitochondrial protein machinery is a target of these antibiotics [52]. The bacterial ribosome-targeting antibiotics, including tetracycline, inhibit bacterial protein translation [53] as well the eukaryotic mitochondrial protein synthesis [54]. Suppressing mitochondrial machinery may have multiple impacts on host fitness. For example, the nematode *Caenorhabditis elegans* and the fly *Drosophila melanogaster* that were fed with tetracycline-supplemented food displayed lower metabolic activity, retarded development, and reduced fertility [52]. Generating and maintaining symbiont-free *B. insularis* is requisite to testing the non-specific antibiotic impacts on host fitness. Other insects, such as the seed-feeding *Riptortus* bug, can be maintained on sterilized soybean seeds for multiple generations [10], providing an avenue to examine axenic insects without antibiotic therapy. Unfortunately, *B. insularis* neonates require live St. Augustinegrass that is known to harbor *Burkholderia*, precluding access to an axenic food source [21].

To generate symbiont-free *B. insularis* without using antibiotics, the lytic BiBurk16MC_R phage that specifically lysed the gut-symbiotic Bi16MC_R_vitro culture was isolated and amplified from soil. Oral administration of BiBurk16MC_R phage to *B. insularis* nymphs had no impact on insect fitness. This bacteriophage was identified as a podovirus and was morphologically similar to other soil-derived lytic phages that target the *B. cepacia* complex (Bcc) species [55,56] and *B. pseudomallei* [57]. Podoviruses have short tails, double-stranded DNA, and occur relatively frequently in *Gammaproteobacteria* and *Bacilli* [58,59]. The 46-kb genome of the BiBurk16MC_R phage, smaller than the 60-kb genome of the Bcc podovirus [55,56], was approximately the same size as 45-kb genome of the *B. pseudomallei* podovirus [57]. Using larvae of *Galleria mellonella* (L) as a model system, the in vivo efficacy of lytic Bcc phages have been tested against the human-pathogenic Bcc strains [60]. A single injection of Bcc phage (2.5 × 10^3^ PFU) rescued 50% of larvae infected with a lethal dose of Bcc bacteria, whereas nearly all of non-injected larvae died [60]. Similarly, injection of environmentally derived *Pseudomonas aeruginosa* phage increased survival time of *P. aeruginosa*-infected *D. melanogaster* [61]. In addition to these laboratory-induced infections, oral delivery of a phage preparation has been reported to suppress the impact of American foulbrood disease caused by *Paenibacillus larvae* in hives of the honeybee, *Apis mellifera* [62].

Even though the previously mentioned studies demonstrated the potential of phage therapy to suppress insect-associated bacteria, oral delivery of *Burkholderia* phages failed to target the crypt-associated *Burkholderia* in vivo in the current study. It should be noted that many, but not all, of the *B. insularis* exposed to orally delivered phage harbored the phage-sensitive bacterial ribotype. Lytic activities were detected in the anterior midgut regions (non-symbiont organs; M1-M3) but not in the posterior regions (symbiont organs; M4B-M4), suggesting that the phage failed to enter the symbiont organ. Similarly, in *R. pedestris*, the non-symbiotic M1 to M3 regions play roles in food storage, digestion, and absorption [63]. When *R. pedestris* nymphs were fed with fluid supplemented with various food dyes and non-symbiotic *E. coli* cells, the dyes and *E. coli*, detected in the M1 to M3 regions, did not enter the symbiont organs (M4B and M4) [63]. A constricted region connecting the non-symbiotic M3 and the symbiotic M4B regions blocked the entry of food materials and other non-*Burkholderia* microbes into the posterior midgut regions [63]. *Blissus insularis* also possesses a thin, connective tissue between the M3 and the M4B regions (see Figure 1C). The failure of orally administrated Evans blue dye and bacteriophage to enter into the M4B to M4 regions is due likely to the observed midgut constriction. As proposed by Ohbayashi et al. [63], after gut symbiont establishment in the symbiont organ (crypts), the constricted region in young coreids and plataspids degenerates and restricts further transport of material into the posterior midgut. This insect modification is reminiscent of the regressed epithelial cells in the bobtail squid, *Euprymna scolopes*, activated by the colonization of its luminous bacterium *Vibrio fischeri* [64,65,66]. Perhaps the closed and degenerated constricted region in many heteropterans is analogous to the regressed surface epithelium of the squid light organ and is induced by the peptidoglycan signals from their Gram-negative bacterial symbionts, as shown in *V. fischeri* [66].

## 5. Conclusions

Our study indicated that antibiotic treatment of fifth-instar *B. insularis* suppressed the levels of viable midgut crypt-associated *Burkholderia*, delayed adult eclosion, and reduced body size of *B. insularis*. Antibiotic-treated *B. insularis* fifth instars exhibited increased susceptibility to bifenthrin. Whether this increased susceptibility was due to the stress induced by either the non-lethal effects of the antibiotics or the elimination of the symbiont is unknown. Lytic phages were successfully isolated from soils and infected the cultured symbiont *Burkholderia*. However, oral administration of phage preparations to symbiont-infected *B. insularis* failed to impact symbiont populations. This failure likely was due to the constriction produced at the junction of the anterior and posterior midgut that prevented phage from entering the crypt region, in which the symbionts densely populate. These results demonstrated an intricate relationship between gut symbionts and *B. insularis*.

## Figures and Tables

**Table 1:** Mean (±SE) values of survivorship, adult eclosion rate, and lytic phage activity of midgut homogenates from *Blissus insularis* that were exposed to different treatments.

Treatment	N *^a^*	% Survivorship	% Adult Eclosion Rate	N *^b^*	% Plaque Formation *^c^*		% Infection Rate (no. Positive/Total) *^d^*
					M1-M3	M4B-M4	
Phage	28	97 (3)	29 (9)	25	80 (8) a	0 (0)	40 (4/10)
Phage + *Burkholderia*	29	100 (0)	18 (7)	25	92 (7) a	0 (0)	50 (5/10)
*Burkholderia*	27	96 (4)	39 (5)	24	0 (0) b	0 (0)	20 (2/10)
Control	26	95 (5)	34 (13)	23	0 (0) b	0 (0)	20 (2/10)
	*χ*^2^ (df) *^e^*	1.1385 (3)	2.3887 (3)		17.1472 (3)	N/A *^f^*	2.2559 (3)
	*P*	0.7678	0.4957		0.0007	N/A	0.5210

*^a^* Number of *B. insularis* fifth instars that were used in each treatment; *^b^* After 10-day exposure to each treatment, the number of surviving *B. insularis* subjected to the lytic phage activity assay; *^c^* The percentage of the *B. insularis* anterior (M1-M3 regions) and posterior (M4B-M4) midgut homogenates producing plaques in the lytic phage activity assay; *^d^* The percentage of M4B-M4 homogenate preparations infected with the target Bi16MC_R_vitro isolate, as detected by BOX-PCR fingerprinting; *^e^* Values of Chi-square and degree of freedom, analyzed by Kruskal-Wallis test. Different letters in the column (% plaque formation M1-M3) indicate statistically significant difference between each treatment (Dwass, Steel, Critchlow-Fligner Method; *p* < 0.05); *^f^* Not available.

## Supplementary Materials

The following are available online at www.mdpi.com/2075-4450/7/4/61/s1, Supplementary File 1.
