# Supplementary material for: Impacts of Antibiotic and Bacteriophage Treatments on the Gut-Symbiont-Associated Blissus insularis (Hemiptera: Blissidae)
Source: Insects. 2016 Nov 3;7(4):61. doi: 10.3390/insects7040061 (PMC5198209; doi:10.3390/insects7040061)
Supplement: Supplementary file 1 [file insects-07-00061-s001.pdf]

insects

Supplementary Materials: Impacts of Antibiotic and Bacteriophage Treatments on the Gut-Symbiont-Associated *Blissus insularis* (Hemiptera: Blissidae)

Yao Xu, Eileen A. Buss and Drion G. Boucias

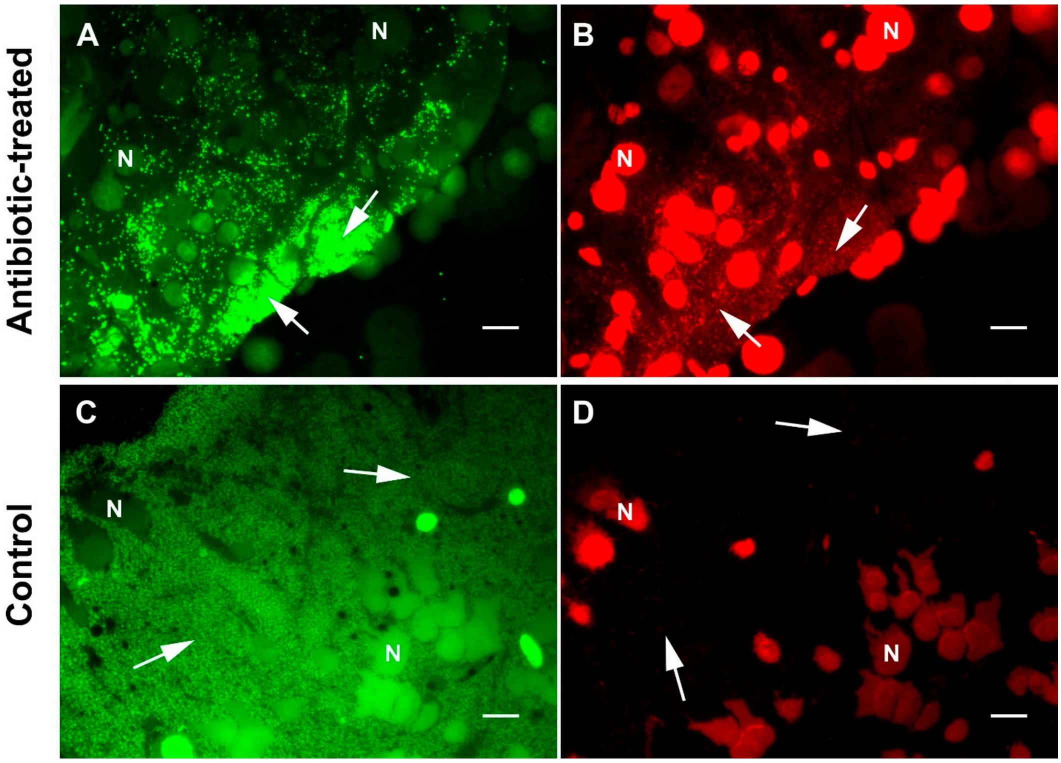

Figure S1. Vital staining of *Blissus insularis* midgut crypts and crypt-inhabiting bacteria. Intact midgut crypts of antibiotic-treated (**A**-**B**) and control (**C**-**D**) *B. insularis* fifth instars stained with the LIVE/DEAD BacLight Kit and observed by using an epifluorescence microscope. Living bacteria fluoresce bright green under blue light (**A** and **C**), while dead bacteria fluoresce red under green light (**B** and **D**). Arrows indicate the stained bacteria. N, host crypt nucleus. Scale bar = 20 µm.

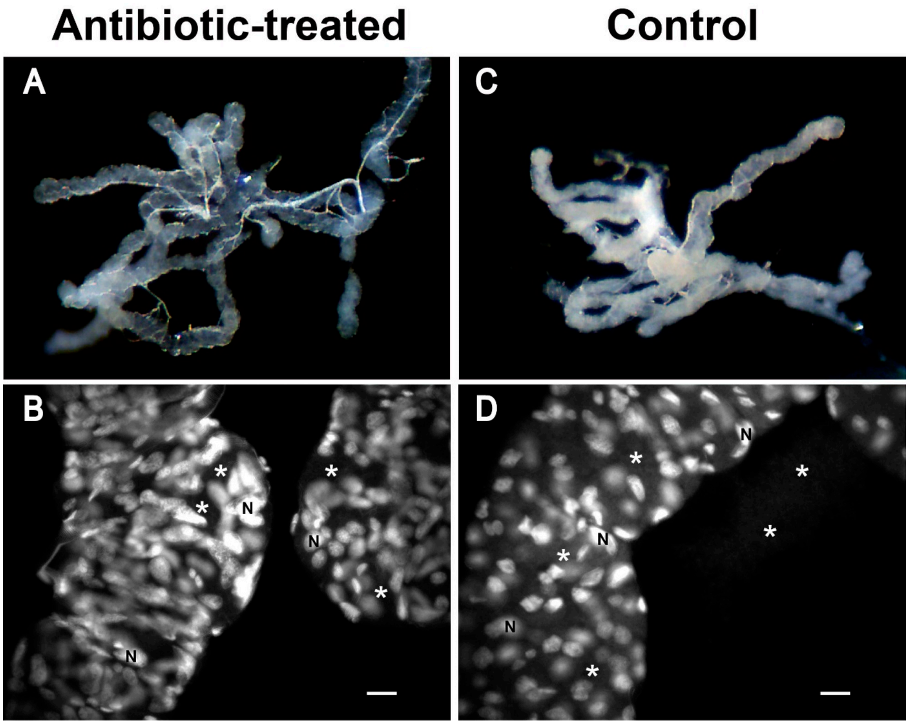

Figure S2. Micrographs of midgut crypts dissected from the antibiotic-treated and control *Blissus insularis* fifth instars. (**A**) Dissected semi-transparent crypts of antibiotic-treated *B. insularis*. (**B**) DAPI-stained crypts of antibiotic-treated *B. insularis*, with a less intense bacterial signal within the crypt lumen (labeled by stars). (**C**) Dissected milky-white crypts of control *B. insularis*. (**D**) DAPI-stained crypts of control *B. insularis*, containing intense bacterial signals within the crypt lumen and the ones that were released from the lumen (labeled by stars). N, host crypt nucleus. Scale bar = 20 μm.

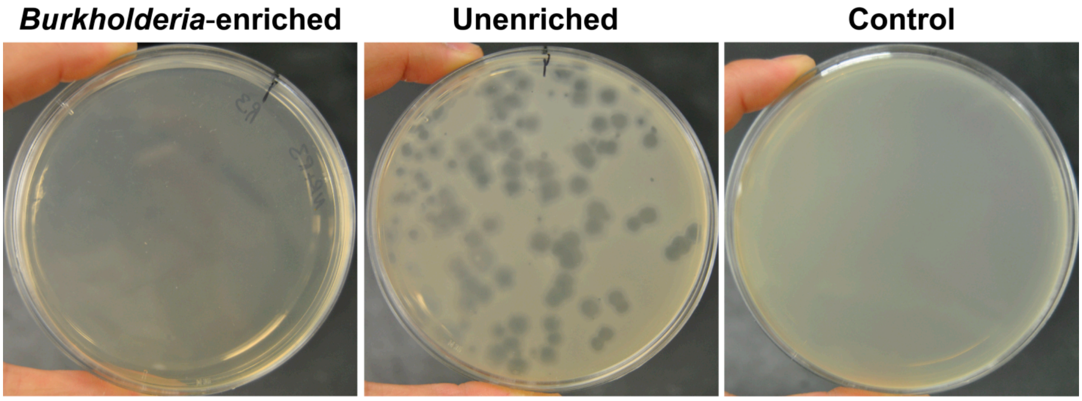

Figure S3. Isolation of gut-symbiotic *Burkholderia* phages from soils using the enrichment method. The *Burkholderia*-enriched soil filtrate cleared its own host bacterial culture (Bi16MC_R_vitro) (left panel), whereas the unenriched soil filtrate produced approximately 100 plaques (middle panel). Control plate included host bacterial culture in the absence of soil filtrate (right panel).

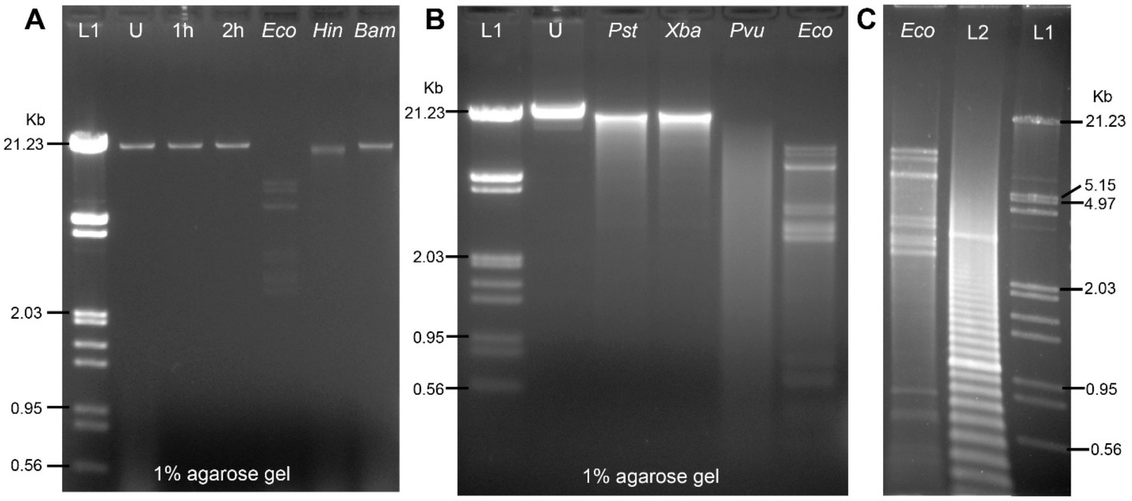

Figure S4. Genomic DNA of a selected gut-symbiotic *Burkholderia* phage (BiBurk16MC_R) digested with six restriction endonucleases. (**A**) 1% agarose gel electrophoresis of undigested phage DNA, Benzonase® nuclease-treated undigested phage DNA for one (1h) and two (2h) hours, and its nuclease-treated for one hour digested patterns by *EcoRV*, *HindIII*, or *BamHI*. (**B**) 1% agarose gel electrophoresis of undigested phage DNA and its digested patterns by *PstI*, *XbaI*, *PvuII*, or *EcoRV*. (**C**) The genome size estimated by PFGE after being digested by the restriction enzyme *EcoRV*. Abbreviations: *Eco*, *EcoRV*; *Hin*, *HindIII*; *Bam*, *BamHI*; *Pst*, *PstI*; *Xba*, *XbaI*; *Pvu*, *PvuII*. Standard markers L1 = Lambda DNA/*EcoRI* plus *HindIII*, L2 = 100-bp molecular ruler.

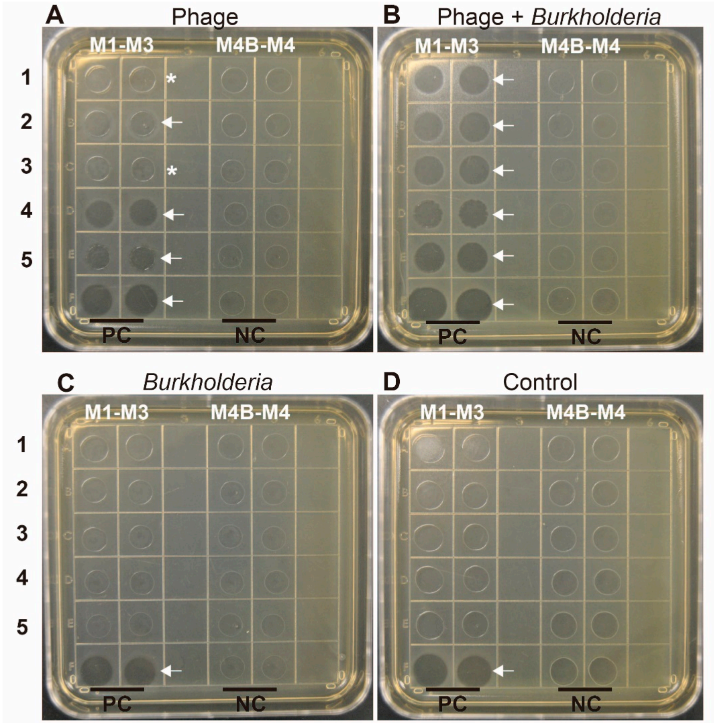

Figure S5. Lytic phage activity assay. Detection of lytic phage activity in *Blissus insularis* midgut homogenates after 10-day exposure to the diet containing *Burkholderia*-specific phage (**A**), phage plus the target *Burkholderia* cells (**B**), *Burkholderia* cells only (**C**), or neither phage nor *Burkholderia* cells (**D**). Rows 1-5 indicated five *B. insularis* individuals for each treatment. Five microliters of homogenate were spotted in duplicate. Arrows indicated the clear zone formation. Stars indicated the formation of small plaques. Abbreviations: M1-M3, homogenates of anterior midgut regions (first to third regions); M4B-M4, homogenates of posterior midgut regions (fourth midgut bulb and fourth midgut crypts); PC, positive control (*Burkholderia* phage BiBurk16MC_R); NC, negative control (TBS buffer).

Table S1. The estimated crypt-associated *Burkholderia* 16S rRNA gene copies from *Blissus insularis* fifth instars fed antibiotic-treated and control food for 10 days.

Treatment	Status [a]	Gender [b]	DNA concentration (ng μL^{-1})	16S rRNA gene copies per insect
Antibiotic-treated (n = 25)	Non-paralyzed	Female	17.3	2.9×10^{7}
	Non-paralyzed	Female	12.1	1.7×10^{7}
	Non-paralyzed	Female	11.2	1.8×10^{7}
	Non-paralyzed	Female	16.2	2.7×10^{7}
	Non-paralyzed	Female	14.6	1.2×10^{7}
	Non-paralyzed	Male	9.3	1.2×10^{7}
	Non-paralyzed	Male	16.2	1.0×10^{7}
	Non-paralyzed	Male	10.1	1.3×10^{7}
	Non-paralyzed	Male	12.8	1.5×10^{7}
	Non-paralyzed	Male	19.7	1.9×10^{7}
	Non-paralyzed	Male	13.1	3.8×10^{7}
	Non-paralyzed	Male	14.7	4.5×10^{7}
	Non-paralyzed	Male	11.1	5.6×10^{7}
	Non-paralyzed	Male	11.8	2.0×10^{7}
	Non-paralyzed	Male	10.2	3.0×10^{7}
	Non-paralyzed	Male	12.2	5.6×10^{7}
	Non-paralyzed	Male	9.3	1.6×10^{7}
	Non-paralyzed	Male	9.3	6.0×10^{6}
	Non-paralyzed	Male	13.4	5.0×10^{7}
	Non-paralyzed	Male	15.6	1.6×10^{7}
	Non-paralyzed	Male	10.4	1.7×10^{7}
	Non-paralyzed	Male	15.0	2.0×10^{7}
	Non-paralyzed	Unknown	8.1	3.8×10^{7}
	Non-paralyzed	Unknown	13.2	1.6×10^{7}
	Non-paralyzed	Unknown	10.5	1.9×10^{7}
			Mean (SE) [c]	$2.1 (0.3) \times 10^{7}$
Antibiotic-treated (n = 13)	Paralyzed	Female	10.9	6.2×10^{6}
	Paralyzed	Female	11.7	3.3×10^{7}
	Paralyzed	Female	11.1	2.5×10^{6}
	Paralyzed	Female	10.5	7.9×10^{6}
	Paralyzed	Female	11.5	9.7×10^{6}
	Paralyzed	Male	8.6	8.1×10^{6}
	Paralyzed	Male	11.8	2.1×10^{7}
	Paralyzed	Male	8.5	1.3×10^{7}
	Paralyzed	Male	12.1	2.7×10^{6}
	Paralyzed	Male	7.8	2.6×10^{6}
	Paralyzed	Male	10.5	1.9×10^{7}
	Paralyzed	Unknown	8.9	2.5×10^{7}
	Paralyzed	Unknown	9.3	4.5×10^{6}
			Mean (SE) [c]	$1.2 (0.3) \times 10^{7}$

Table S1. *Cont.*

Treatment	Status [a]	Gender [b]	DNA concentration (ng μL^{-1})	16S rRNA gene copies per insect
	Non-paralyzed	Female	16.0	7.9×10^{7}
	Non-paralyzed	Female	12.7	9.1×10^{7}
	Non-paralyzed	Female	16.1	1.0×10^{8}
	Non-paralyzed	Female	30.8	1.8×10^{8}
	Non-paralyzed	Female	21.6	2.0×10^{8}
	Non-paralyzed	Female	28.9	4.1×10^{8}
	Non-paralyzed	Female	22.1	1.8×10^{8}
	Non-paralyzed	Female	32.5	3.8×10^{8}
	Non-paralyzed	Female	23.8	4.7×10^{8}
Control	Non-paralyzed	Female	13.7	1.1×10^{8}
(n = 20)	Non-paralyzed	Female	30.1	1.5×10^{8}
	Non-paralyzed	Male	15.2	1.4×10^{8}
	Non-paralyzed	Male	15.5	1.8×10^{8}
	Non-paralyzed	Male	19.7	2.8×10^{8}
	Non-paralyzed	Male	18.1	2.0×10^{8}
	Non-paralyzed	Male	13.6	2.2×10^{8}
	Non-paralyzed	Unknown	12.1	1.1×10^{8}
	Non-paralyzed	Unknown	20.6	1.8×10^{8}
	Non-paralyzed	Unknown	8.9	5.1×10^{7}
	Non-paralyzed	Unknown	11.9	1.1×10^{8}
			Mean (SE) [c]	$1.9 (0.3) \times 10^{8}$
	Paralyzed	Female	9.1	4.8×10^{7}
Control	Paralyzed	Female	15.5	2.9×10^{8}
(n = 4)	Paralyzed	Male	10.1	5.5×10^{7}
	Paralyzed	Male	10.7	7.6×10^{7}
			Mean (SE) [c]	$1.2 (0.5) \times 10^{8}$

[a] The status of fifth instars that were exposed to 0.1 μg·mL^{-1} of bifenthrin for 24 hours. [b] Gender was assessed during dissection, based on the presence of the internal gonadal tissue. Unknown means the sample whose internal gonadal tissue was destroyed accidentally during dissection. Only 53 fifth instars were able to be sexed. [c] Mean and standard error were calculated within each status column. See statistical analyses in Figure 3.

Table S2. Spot-on-the-lawn assay of soil filtrates enriched with different cultured symbiont *Burkholderia* isolates, tested with their host bacterial lawns and heterogeneous *Burkholderia* lawns.

**Lawn identity**	**Soil filtrates enriched with cultured *Burkholderia* isolates**										
	**Bi12 MC_S**	**Bi14 MC_S**	**Bi16 MC_S**	**Bi20 MC_S**	**Bi21 MC_S**	**Bi26 MC_S**	**Bi16 MC_R**	**Bi17 MC_R**	**Bi20 MC_R**	**Bi22 MC_R**	**Bi24 MC_R**
Bi12MC_S	++ *	++	-	-	-	-	-	-	-	-	-
Bi14MC_S	++	++ *	-	-	-	-	-	-	-	-	-
Bi16MC_S	-	N/A	++*	-	+	-	+	-	-	++	++
Bi20MC_S	-	N/A	-	-	-	-	-	-	-	-	-
Bi21MC_S	-	N/A	-	-	-	-	-	-	-	-	++
Bi26MC_S	-	N/A	-	-	-	-	-	-	-	-	-
Bi16MC_R	-	N/A	-	-	++	+	++*	-	-	-	-
Bi17MC_R	-	-	-	-	-	-	-	-	-	-	-
Bi20MC_R	-	N/A	-	-	-	-	-	-	-	-	-
Bi22MC_R	-	-	-	-	-	-	-	-	-	++*	-
Bi24MC_R	-	N/A	-	-	-	-	-	-	-	-	-

++ clear zone (7-9 mm in diameter); +, 10-15 small plaques; -, no plaque; N/A. not available. * The *Burkholderia* phage that was enriched in the host bacterial culture homogeneously targeted on its host bacterial lawn.

© 2016 by the authors. Submitted for possible open access publication under the terms and conditions of the Creative Commons Attribution (CC-BY) license (http://creativecommons.org/licenses/by/4.0/).
